# Theoretical Models and Operational Frameworks in Public Health Ethics

**DOI:** 10.3390/ijerph7010189

**Published:** 2010-01-18

**Authors:** Carlo Petrini

**Affiliations:** Italian National Institute of Health, Office of the President, Bioethics Unit, Via Giano della Bella 34, I-00162 Rome, Italy; E-Mail: carlo.petrini@iss.it; Tel.: +39-06-4990-4021; Fax: +39-06-4990-4303

**Keywords:** ethics, public health, utilitarianism, personalism, foundations, models, frameworks

## Abstract

The article is divided into three sections: (i) an overview of the main ethical models in public health (theoretical foundations); (ii) a summary of several published frameworks for public health ethics (practical frameworks); and (iii) a few general remarks. Rather than maintaining the superiority of one position over the others, the main aim of the article is to summarize the basic approaches proposed thus far concerning the development of public health ethics by describing and comparing the various ideas in the literature. With this in mind, an extensive list of references is provided.

## 1. Clinical Ethics and Public Health Ethics

Clinical practice is characterized by a personal physician-patient relationship [1]. Conversely, public health practice is characterized by global attention to whole populations and therefore by an emphasis on collective health conditions, prevention, and social, economic, and demographic determinants of health and disease [2]. The collective perspective is described effectively by the Institute of Medicine (IOM) in its well-known report, “The Future of Public Health,” in which public health is defined as “what we, as a society, do collectively to assure the conditions for people to be healthy” [3].

The historical relationship between clinical ethics and public health ethics is the subject of debate: differing and sometimes contradicting opinions regarding both the historical development of public health ethics and its main cultural models can be found throughout the literature.

Some authors suggest that public health ethics, in addition to its slower and more recent historical development, is actually in conflict with clinical ethics: “in contrast to the traditional emphasis of bioethicists on the physician-patient relationship, public-health ethics focuses on the design and implementation of measures to monitor and improve the health of populations. In addition, public-health ethics looks beyond health care to consider the structural conditions that promote or inhibit the development of healthy societies” [4]. Most of the authors ascribe this difference to the utilitarian nature of public health. For example, Bayer and Fairchild attribute the origins of this discrepancy to the eminently utilitarian approach of public health, which is aimed at maximizing collective well-being even to the detriment of individual care [5].

On the contrary, Wynia and other authors believe that public health ethics is neither slow [6] nor opposed [7] to clinical ethics and that new “bridges between medical care and public health” should be built [8].

There are also middle positions. For example, Dozon and Fassin have studied the historical development of public health with “an anthropological approach”: even though the authors do not explore the historical development of public health ethics in great depth, they highlight the cultural development of public health and include “morality” in this discussion [9].

In this sometimes debated area, it is highly significant to note the absence of chapters devoted specifically to public health ethics in the majority of books on the history of bioethics and medical ethics [10–16]. Similarly, books discussing the theoretical basis of bioethics usually have not discussed the foundations of public health ethics until recent years. This characteristic seems to be present in books from various cultural and linguistic contexts, including English [17], French [18], and Italian [19].

On the other hand, the historical perspective on the ethics of public health is not disregarded in public health texts [20,21]. This situation is meaningful: it indicates that while bioethics has not paid much attention to public health ethics for many years, public health professionals themselves perceive a real need for it.

Regardless of potentially different interpretations of its historical development, interest in public health ethics is undoubtedly growing on at least three levels: operational [22], deontological [23], and theoretical [24]. In other words, if there was actually a delay in the emergence of public ethics with respect to clinical ethics during the early years in which bioethics began to become consolidated as an autonomous field of study during the seventies and eighties, then during the nineties that gap was closed. This evolution has been pointed out by Kass, who published a historical analysis of public health ethics in 2004, subdividing it into two main periods: “The early era: 1970s−1990s” and “Stage II: the emergency of frameworks, the language of public health ethics” [25].

Since the nineties, the increasing importance of ethical issues in the debates about public health policies has become evident in different cultural contexts. Examples in francophone countries include the Ethics and Public Health (*Éthique et santé publique*) Conference that took place in Nantes on March 13–14, 1997 [26] and the National Public Health Priorities (*Priorités nationales de santé publique*) Conference that took place in Montréal on November 18–19, 1997 [27]. Many aspects of public health ethics have been extensively developed in anglophone countries. Among others, one example is the ethics of infectious diseases [28]: Dawson and Verweij have provided important contributions to this field [29].

## 2. Cultural and Philosophical Models in Public Health Ethics

### 2.1. Different Assessments

There are evident differences not only regarding the relationship between clinical and public health ethics, as pointed out in the previous section, but also regarding the assessment of the primary cultural and philosophical models grounding public health ethics. For example, according to Coughlin, some “approaches to moral reasoning, such as . . . duty-based theories . . . and communitarianism have not been widely applied in public health” [30]. Yet on the contrary, having analyzed public health ethics theories, Beauchamp and Steinbock maintain that Kantian moral theory, which is the main duty-based theory, “has been dominant in both moral thinking and *public policies*” [author’s italics] and that “communitarianism is particularly appropriate for the aggregate approach to policy exemplified by public health” [31].

### 2.2. The Utilitarian Roots of Public Health and the Conflicts between Individual and Social Interests

Several authors have also pointed out that great importance has been afforded to autonomy—and therefore to issues such as informed consent, confidentiality, and so forth—in contemporary bioethics, to the point that it has become at least a *primus inter pares* if not a clearly superseding principle. The principle of autonomy has tended to dominate healthcare ethics especially in North America [32]. On the contrary, however, public health is based predominantly on population-level utility, making it more attentive to issues such as epidemics, social determinants of health, and cost-effective decision making: a “pervasive utilitarian component” in public health is thereby “undeniable” [33].

This utilitarian approach is often connected to the question of an alleged paternalism in public health: many philosophers have seen the principal issue of public health as that of paternalism, or the intrusion of the State upon individual liberty in order to promote health and safety. Consequently, as indicated already in Section 1, most ethical problems in public health are characterized by tension between private or individual interests and public or social interests. The main challenge lies embedded in the “relationship between individual and population health” [34]: many authors “have highlighted important features that differentiate public health ethics from bioethics, especially public health’s emphasis on population health rather than issues of individual health” [35].

The utilitarian approach underlying public health, however, is not necessarily synonymous with a lack of attention to individual needs: according to Mackenbach, “the large-scale altruism of public health has to be balanced with the value of individual autonomy, and . . . some degree of dreaming of a better and healthier world is indispensable for further progress in public health . . . The ethical foundations of public health are not always self-evident, and . . . critical reflection on these foundations was, is, and will always be necessary” [36]. Unfortunately, Mackenbach’s “cherished approach is an inherently ‘idealistic’ one” [37].

In the introduction to the book *Public Health Policy and Ethics*, Boylan describes the tension between private and public interests and identifies a distinction between “prudential grounds” and “moral grounds” for public health. “The prudential model is based upon a principle of selfish egoism and extended egoism (the political expression of selfish egoism),” and the author suggests that “moral grounds for public health are more certain because they give a clear and intersubjective foundation” [38]. The question we must address, then, is the following: what lies at the roots of public health ethics?

### 3. Models for Public Health Ethics

Classical *utilitarianism* was formulated in the 19^th^ century by Jeremy Bentham [40] and John Stuart Mill [41]. According to utilitarianism, actions are right insofar as they tend to promote the greatest happiness for the greatest number, and wrong as they tend to promote the opposite [42]. Utilitarianism is therefore a form of consequentialism: not all consequentialists are utilitarians, but all utilitarians are consequentialists. Utilitarianism is a maximizing theory: right actions and policies are those that achieve the greatest happiness possible.

The problem of “sentience” is important in every ethical theory. It is of particular importance in the utilitarian model since most utilitarians consider the ability to experience pain and pleasure an important element for assessing utility.

Many contemporary ethicists and philosophers are in line with utilitarian theories: utilitarian theories seem to be an effective way of maximizing benefits for the greatest number of people. Nevertheless, there are many situations in which maximizing happiness could conflict with other values, namely justice, fairness, and honesty. Objections against utilitarianism point to its intrinsic injustice, since this theory only considers the amount of good but not the way in which it is distributed. Moreover, all benefits cannot be measured according to a single standard, especially where money is involved. For example, improvements in health conditions cannot be measured in the same way as saving or extending life.

According to the *deontological theories*, the good is known by its consistency with moral rules and principles. Kant’s theory is the best known example of deontological theories. Kant emphasizes the connection between reason and morality: reason, according to Kant [43], is what separates human beings form the rest of the animals and what makes man subject to the moral law; since man is a moral agent, he is responsible for his actions. Kantian ethics objects to consequentialism; however, this does not mean consequences can or should be ignored [44]. Consequences become relevant only if the proposed actions are morally permissible: according to Kant, actions are intrinsically right or wrong regardless of their consequences. When we want to know if a proposed action is morally permissible, the question we must therefore ask ourselves is not about the likely consequences of doing the act; rather, the guiding principle of action should be to “act only on the maxim of an action that you consistently will universally” [45].

*Communitarian ethics* rejects the notion of timeless, universal, ethical truths based on reason [46]. According to communitarian theories, morality is a cultural rather than abstract concept. Communitarians maintain that our moral thinking has its origins in the historical traditions of particular communities. Communities are not simply collections of individuals: they are groups of individuals who share values, customs, institutions, and interests. Communitarian ethics seeks to promote the “common good” in terms of shared values, ideals, and goals. In the communitarian perspective, the health of the public is one of those shared values: reducing disease, saving lives, and promoting good health are shared values [47,48].

Communitarian ethics has been criticized on both practical and moral grounds. One of the problems with communitarian ethics, like utilitarianism, is that the vision of what constitutes a “good life” may differ: therefore, there is an inherent risk of a “tyranny of the majority.” Community health programs may involve selection of benefit structures that favor some citizens over others. Taken to its extreme, the communitarian viewpoint—by making even universal values subject to a community filter—could threaten the sense of a common humanity and undermine political and social cooperation.

*Egalitarian theories* typically stress equal access to certain goods, but not equal sharing of all possible social benefits. John Rawls explains his theory of equal opportunities with the metaphor of how a rational agent behind an objective veil of ignorance would choose principles of justice [49]. Rawls applied his theories of justice to health care only in later works [50]. Other authors, however, and especially Daniels [51], have employed his theories to propose public health models providing equal opportunities. This approach emphasizes the need for fair procedures to be used in solving problems of rationing and conflicts between individual and social interests in public health.

From a practical perspective, critics consider this model insufficient to address the need for efficiency, willingness to pay, and other problems. From a theoretical perspective, the model does not seem to adequately determine goods from which no one can be excluded and values other than equity. Other concerns about the equal opportunity model include the exclusive focus on means and resources, thereby neglecting ends, and its inattention to individual differences and social peculiarities [52].

*Liberalism* stresses equal access to rights and free-market based approaches. The predominant values espoused are therefore individual freedom and autonomy. According to liberalists, the role of public authorities is to protect individual rights, and the state should maintain a neutral position with respect to the various understandings of good [53]. Unlike the libertarians, liberalists claim that human well-being requires a certain amount of positive rights and corresponding duties. Critiques of liberalism stress that health care is different from economics and is not able support the conditions for market allocation [54].

*Contractualist theories* consider fair and morally right decisions to be based on procedural justice and open processes whereby citizens are involved in the deliberations. This approach requires criteria for decision making to be clearly settled in advance [55]. Several critiques of this models have been expounded. Some authors indicate how theories of just processes ignore deeper and more fundamental moral questions. Moreover, contractualists theories can never be universal or unbounded by culture [56].

*Personalism* considers the individual to be the core value and tries to achieve the common good by promoting and enhancing the good of the individual. The main values proposed by personalism include respect for life (public health actions are aimed at protecting and promoting human life and health), sociality and solidarity (social solidarity means and involves a commitment to bridge the gap between the different sectors of society and to integrate them into a community), and responsibility (the responsibility to prevent and protect against avoidable diseases, the duty not to create irresponsible burdens for the society, and responsibility for people in need) [57,58]. Personalism has been criticized since terms “person” and “personalism” have myriad uses: there are atheistic, idealistic, Christian and other personalisms [59].

In *casuistry*, decision making takes place at the level of the particulars of the case itself. Evaluations are not referred to a particular theory; rather, maxims are identified that have a bearing on the case. Maxims are simply rules such as “tell the truth” or “be compassionate.” Casuistry requires clearly expounding the facts. Decisions are then made on the basis of the most appropriate maxims for the specific circumstances [60].

Other classifications of the models are also possible. For example, Häyry suggests “three major ethicopolitical approaches to all public activities.” He summarizes the various models into three categories: welfare liberalism, traditional communitarianism, and radical libertarianism. The author identifies a list of words that highlight the main concepts of each approach. For welfare liberalism, they include autonomy, nonmaleficence, beneficence, justice, privacy, consent, confidentiality, and others. For traditional communitarianism, they include integrity, vulnerability, solidarity, subsidiarity, social democracy, honesty, respect, and others. For radical libertarianism, they include liberty, general happiness, non-violation of rights, voluntariness, other people’s interests, non-interference, contract and compensation, and others. According to the author, however, “it seems that public health arguments do not deal with all the concerns that people have” [61].

Many philosophers have seen the principal issue of public health as that of paternalism, or the intrusion of the state upon individual liberty in order to promote health. These ethical models show that the dispute is far more extensive than the debate over paternalism [62]. Our aim here is not to determine the best moral theory, but to show that all of them have something to contribute to the debate.

## 3. Public Health Ethics in Practice: Examples of Ethical Frameworks

Public health ethics is not only in need of theoretical models, but also of practical frameworks. Unlike the duties of clinicians to patients in clinical medicine, professional standards for ethical practice are not well defined in public health. As Taboada and Cuddenback observe, “incorporating ethical analysis into public health raises many challenging questions. For example, what does ethical analysis add to public health beyond legal or public policy analysis? Is the law itself subject to a process of ongoing ethical scrutiny? When ethicists appeal to ‘values,’ who gets to decide which values are worthy of protection or how these values should be prioritized in cases of conflict? How should ethical analysis address the tension between universal principles and culturally specific values, and find common ground among individuals from diverse cultural backgrounds? Such questions have practical implications for how public health policies are designed, implemented, and evaluated” [63].

In 2001 Kass published “an analytic tool, designed to help health professionals consider the ethics implications of proposed interventions, policy proposals, research initiatives and programs” [64]. The tool is based on six main questions: (1) “what are the public health goals of the proposed program?”; (2) “how effective is the program in achieving its stated goals?”; (3) “what are the known or potential burdens of the program?”; (4) “can burdens be minimized . . . [and] are there alternative approaches?”; (5) “is the program implemented fairly?”; and (6) “how can the benefits and burdens of the program be fairly balanced?”.

In 2002, Childress and colleagues suggested some “general moral considerations” for an ethical framework in public health ethics: producing benefits; avoiding, preventing, and removing harms; producing the maximal balance of benefits over harms and other costs (often called utility); distributing benefits and burdens fairly (distributive justice); ensuring public participation, including the participation of affected parties (procedural justice); respecting autonomous choices and actions, including liberty of action; protecting privacy and confidentiality; keeping promises and commitments; disclosing information as well as speaking truthfully (often grouped under transparency); and building and maintaining trust [65].

In 2006, Nieburg, Bernheim Gaare and Bonnie proposed a “Guide to considering the ethical issues in public health practice.” The “guide” is composed of a list of questions subdivided in four main areas: “Assessing public health practice,” “Identifying and recognizing ethical issues and considerations,” “Identifying options and making and implementing public health decisions,” and “Later: evaluating the resolution(s)” [66].

In 2007, Baum and co-authors observed that “public health practitioners may lack the experience, time, resources (including training), or even motivation to deliberately consider ethics in their daily wok . . . However, we believe that greater clarification of the ethical underpinnings of their decisions can add value to public health works for several reasons. First, ethical clarification ensures that officials and practitioners unmask normative assumptions and explicitly, rather than implicitly, analyze values during their assessments . . . Second, ethical clarification helps balance economic analysis . . . Third, clarifying the ethical considerations integrated into a particular policy or program decision can help to illuminate decision-makers’ conceptions of the appropriate scope of public health practice.” On this basis, they suggested a framework composed of six steps: (1) “determine population-level utility of the proposed action,” (2) “demonstrate evidence of need and effectiveness of actions,” (3) “establish fairness of goals and proposed implementation strategies,” (4) “demonstrate accountability,” (5) “assess expected efficiencies and costs associated with the proposed action,” and (6) “consider political feasibility and community acceptance” [67].

Two examples that seem particularly exhaustive are described here in greater detail than those mentioned above: the Public Health Code of Ethics, by the Public Health Leadership Society (PHLS) and the American Public Health Association (APHA), and the EuroPHEN project.

In 2002, the PHLS proposed the Public Health Code of Ethics consisting of 12 principles [68]. The Code is accompanied by guidelines [69], and has also been adopted by the APHA [70]. Principle 1 identifies the primary goal of public health, which is to address “the fundamental causes of disease and requirements for health, aiming to prevent adverse health outcomes.” This aim is strengthened by principle 5, which refers to “policies and programs that protect and promote health”. In principle 2, the PHLS emphasizes the need for public health policies and community interests to respect the “rights of individuals in the community.” The PHLS recommends that communities be informed about public health policies (principle 6), and information is considered a requirement for effective and timely (principle 7) involvement of community members in the development of health policies (principle 3). Fairness, justice, equity are other basic principles in public health ethics (principle 4). Moreover, public health decisions should be based on scientifically sound information (principle 5), which is also the basis for timely intervention (principle 7) and improvement of the physical and social environment (principle 9). Other ethical requirements include transparency (principle 12), professional competence (principle 11), and personal data protection (principle 10). The PHLS also gives special attention to “diverse values, beliefs, and cultures in the community”: respect for them is a duty (principle 8).

According to the PHLS and the APHA, the following values and beliefs are crucial assumptions inherent to the public health perspective underlying the 12 principles of ethical public health practice:

- “Humans have a right to the resources necessary for health”
- “Humans are inherently social and interdependent”
- “The effectiveness of institutions depends heavily on the public’s trust”
- “Collaboration is a key element to public health”
- “People and their physical environment are interdependent”
- “Each person in a community should have an opportunity to contribute to public discourse”
- “Identifying and promoting the fundamental requirements for health in a community are a primary concern to public health”
- “Knowledge is important and powerful”
- “Science is the basis for much of our public health knowledge”
- “People are responsible to act on the basis of what they know”
- “Action is not based on information alone”

From 2003 to 2006, the European Public Health Ethics Network (EuroPHEN) carried out a project entitled “Public policies, law and bioethics: A framework for producing public health policy across the European Union.” EuroPHEN’s aim was to develop a framework for producing common approaches to public health policy across Europe. EuroPHEN compared the organization of public health structures and public policy responses to selected public health problems in member states to examine how public policy in different countries weighs competing claims of private and public interest [71]. In the analysis performed within EuroPHEN, several requirements for a public health ethical framework were highlighted. First of all, it is important to be aware that a community is not a homogenous whole and to recognize that there are different cultures and disenfranchised members within the community. Next, it must be recalled that

- “Public health institutions should respect the confidentiality of information that can bring harm to an individual or community if made public.”
- “In general consent must be given by the individual before giving any treatment or care, and competency is an important element of informed consent.”
- “Population communication strategies need to be comprehensive to meet the very different needs of all members of the population.”
- “Clinical professional codes tend to stress the need to respect diversity and not discriminate regarding patients or colleagues on the basis of a range of items (personal beliefs, religion, nationality, race, political affiliation, gender, ethnicity, age, socioeconomic grouping or patient disability). At the level of public health policy, certain of these items which are connected to health may be the basis for positive discrimination (age, socio-economic group, patient disability).”
- “Public health ethical codes could contain a requirement to treat people with respect and consideration for dignity, privacy, *etc*. at a population level. Respect for dignity and integrity should not be seen as implying that a public health professional must do everything that an individual or even what the majority of a population may want. Rather their interests should be considered along with the interests of other individuals and groups in the population.”
- “Public health policy should be implemented in a transparent manner that facilitates accountability, including the provision of all information and evidence used to inform the decision making process.”
- “There should be trust in a public health professional to protect and promote the well-being of the population as a whole.” Careful policies to avoid conflicts of interest should therefore be adopted.
- “Public health professionals” are expected “to identify and minimize risks for a population, just as clinicians are required to minimize risk to patients and clients.”
- For a public health professional, prioritization and resource allocation decisions are unavoidable. “Sometimes public health professionals become involved in making decisions, when they must discriminate between the interests of individuals, e.g., in communicable disease control. However, the important ethical issue is that this discrimination is fair and equitable. Similarly situated individuals should have equal access to health care services”.

These frameworks can be applied in multiple ways. They can be used to assess areas of ethical tensions in practice and to provide ways in which to deliberate. The frameworks are conceived to be sufficient flexible for use in practice and to balance several competing considerations rather than setting priorities among principles. Rather than emphasizing a particular ethical principle to guide decision-making, they suggest possible processes by which decisions could be reached and evaluated.

## 4. Conclusions

Three main kinds of questions can be raised in every ethical analysis: descriptive, theoretical, and normative. Descriptive ethics seeks to answer the question “what is right and good?”. It examines how ethics is actually expressed and put into practice. Theoretical ethics seek to answer the question “how do we justify our judgments about the right and the good?”. It is therefore concerned with justifications. Since theoretical ethics discusses what is good and what makes decisions good, its focus is on philosophical—not empirical—questions. Normative ethics focuses mainly on the arguments for how we ought to choose the good and act rightly.

The various schools of ethics (Section 2) tend to emphasize one question over the others, but all three questions are involved to some extent in any ethical theory [72]. Some of the ethical models are very different and irreconcilable, whereas certain aspects present conflicts that are not so definitive. For example, Childress and Bernheim Gaare suggest moving “beyond the liberal and communitarian impasse” [73].

According to the author of this contribution, personalism is the best approach to face ethical problems not only in clinical bioethics, but also in public health ethics [74]. Personalism, which suggests building up the common good on the basis of attention to and care for the good of each person, is the best way to solve conflicts between individual interests and social interests [75]. However, as stated earlier, the aim of this contribution is not to maintain the superiority of one model over the others, but to provide an overview of the models proposed in the literature.

In this light, Raymond Massé suggested a model that can be mentioned as a conclusion. The author identifies a list of widely shared “lighthouse values” [76], including respect for life, beneficence, the common good, responsibility, justice, solidarity, nonmaleficence, autonomy, privacy, utility, and precaution [77]. The author also suggests these principles as a basis for public health legislation [78].
